# Fluid biomarkers in Alzheimer’s disease – current concepts

**DOI:** 10.1186/1750-1326-8-20

**Published:** 2013-06-21

**Authors:** Christoffer Rosén, Oskar Hansson, Kaj Blennow, Henrik Zetterberg

**Affiliations:** 1 Department of Psychiatry and Neurochemistry, Institute of Neuroscience and Physiology, The Sahlgrenska Academy at the University of Gothenburg Mölndal, S-431 80, Mölndal, Sweden; 2 Department of Clinical Sciences Malmö, Clinical Memory Research Unit, Lund University, Malmö, Sweden; 3 UCL Institute of Neurology, London, UK

**Keywords:** Alzheimer’s disease, Cerebrospinal fluid, Blood, Biomarker, Amyloid β, Total tau, Phosphorylated tau, Diagnosis, Disease stages

## Abstract

The diagnostic guidelines of Alzheimer’s disease (AD) have recently been updated to include brain imaging and cerebrospinal fluid (CSF) biomarkers, with the aim of increasing the certainty of whether a patient has an ongoing AD neuropathologic process or not. The CSF biomarkers total tau (T-tau), hyperphosphorylated tau (P-tau) and the 42 amino acid isoform of amyloid β (Aβ42) reflect the core pathologic features of AD, which are neuronal loss, intracellular neurofibrillary tangles and extracellular senile plaques. Since the pathologic processes of AD start decades before the first symptoms, these biomarkers may provide means of early disease detection. The updated guidelines identify three different stages of AD: preclinical AD, mild cognitive impairment (MCI) due to AD and AD with dementia. In this review, we aim to summarize the CSF biomarker data available for each of these stages. We also review results from blood biomarker studies. In summary, the core AD CSF biomarkers have high diagnostic accuracy both for AD with dementia and to predict incipient AD (MCI due to AD). Longitudinal studies on healthy elderly and recent cross-sectional studies on patients with dominantly inherited AD mutations have also found biomarker changes in cognitively normal at-risk individuals. This will be important if disease-modifying treatment becomes available, given that treatment will probably be most effective early in the disease. An important prerequisite for this is trustworthy analyses. Since measurements vary between studies and laboratories, standardization of analytical as well as pre-analytical procedures will be essential. This process is already initiated. Apart from filling diagnostic roles, biomarkers may also be utilized for prognosis, disease progression, development of new treatments, monitoring treatment effects and for increasing the knowledge about pathologic processes coupled to the disease. Hence, the search for new biomarkers continues. Several candidate biomarkers have been found in CSF, and although biomarkers in blood have been harder to find, some recent studies have presented encouraging results. But before drawing any major conclusions, these results need to be verified in independent studies.

## Introduction

The diagnosis of Alzheimer’s disease (AD) can only be made with certainty after a patient has deceased, by histological examination of brain tissue at autopsy or, rarely, following brain biopsy. Histopathology should reveal evidence of the characteristic hallmarks of AD, which are extracellular accumulations of amyloid β (Aβ) in senile plaques and intracellular neurofibrillary tangles of hyperphosphorylated tau (P-tau) [1]. These hallmarks must be seen in sufficient numbers to confer a pathological diagnosis. This fits with the idea of a gradual progression and continuum of pathology and biomarker changes. Multiple pathologies may also be present, and the level of each can be critical.

In 1984, a working group established by the National Institute of Neurological and Communicative disorders and Stroke and the Alzheimer’s Disease and Related Disorders Association created clinical diagnostic guidelines of probable AD [2], which were validated against neuropathological diagnosis with a sensitivity and specificity of around 80 and 70%, respectively [3]. However, the criteria only allowed for making a diagnosis in the dementia stage of the disease, i.e., when the patients were so severely affected by the disease process that they could not manage daily functioning in respect to intellectual and social abilities. Due to advances in research a revision of the guidelines was made by a new working group from the National Institute of Aging in 2011 [4]. Now, imaging and cerebrospinal fluid (CSF) biomarkers have been implemented to provide evidence of an ongoing AD pathophysiological process and it is also possible to make a pre-dementia diagnosis of AD. The included CSF biomarkers are the total amount of tau (T-tau), which reflects the intensity of neuroaxonal degeneration, P-tau, which may correlate with tangle pathology, and the 42 amino acid isoform of Aβ (Aβ42), which correlates inversely with plaque pathology [5]. It is well known that the pathological processes in the brains of AD patients start more than a decade before the first symptoms are noticed [6]. The temporal dynamics of biomarker levels in relation to changes in cognition have been described in a hypothetical model on the continuum of AD [7]. In line with this, the revised diagnostic guidelines identify three different stages of AD: preclinical AD, mild cognitive impairment (MCI) due to AD and AD with dementia. In this review, we will describe the CSF biomarker characteristics of these three stages, and also give an update on the available data for biomarkers in blood. Imaging biomarkers will not be covered.

### Biomarkers in preclinical AD

The working group of the National Institute of Aging was posed with the task of how to define the preclinical phase of AD [8]. They subdivided this phase into three separate stages. In the first stage, patients have amyloid pathology, as defined by positive amyloid on positron emission tomography (PET) imaging or low CSF Aβ42, but no signs of neuronal degeneration (normal volumetric magnetic resonance imaging [MRI] of the brain, normal CSF T-tau levels). In the second stage, neuronal degeneration is evident and patients have elevated CSF T-tau or signs of neuronal injury on imaging methods in addition to positive amyloid markers. To qualify in the third stage, patients have to experience subtle cognitive deficits, although not severe enough to meet the criteria for MCI. To investigate the practical applicability of these guidelines, Jack et al. applied them to a set of cognitively normal individuals from a population-based sample [9]. Only imaging, not fluid, biomarkers were used in this study. After evaluating the results, they found the need for two additional stage categories: stage 0 and Suspected non-AD pathophysiology (SNAP). Stage 0 denotes individuals with no pathological AD biomarkers and no signs of subtle cognitive impairment. Subjects with SNAP have normal amyloid biomarkers, but display signs of neurodegeneration, and could be in the preclinical stage of a non-AD neurodegenerative process. The proposed criteria are so far intended for research purposes only, and can provide a common ground to facilitate the search for biomarkers for preclinical AD. If a disease-modifying treatment for AD is found, it will most likely have the greatest effect if administered in the early disease stages [10], which underlines the importance of the work executed by this working group.

Both cross-sectional and longitudinal studies have evaluated the association between the core CSF AD biomarkers and preclinical AD. Their findings are summarized in Tables 1 and 2. The cross-sectional studies have included patients carrying familial AD (FAD)-causing mutations and determined biomarker levels long before expected disease onset. Bateman et al. evaluated biomarkers levels in subjects at risk for carrying an autosomal dominant AD mutation [11]. The expected age of symptom onset was calculated from the participants’ parents’ age at onset of AD symptoms. Linear models were then created, showing the biomarker levels as a function of time to expected onset of symptoms in mutation carriers and non-carriers. Mutation carriers had significantly elevated levels of CSF T-tau and plasma Aβ42 15 years before expected symptom onset. CSF levels of Aβ42 were significantly reduced in mutation carriers 10 years before expected onset of symptoms. Another study that included members of the Colombian Alzheimer’s Preventive Initiative Registry found that CSF and plasma Aβ42 levels in young individuals carrying a specific presenilin 1 (PS1) mutation were significantly increased compared to non-carriers more than two decades before estimated MCI onset [12]. Studies on cognitively normal mutation carriers that were closer to the expected onset of AD have found decreased levels of Aβ42 [13,14], increased levels of T-tau and P-tau [13,15], and reduced Aβ42:40 ratio in CSF or increased plasma Aβ42 levels [15] compared with controls. These studies are thought to constitute models that are applicable to patients with sporadic AD as well.

**Table 1:** Summary of preclinical studies on patients with familial AD

**Familial AD**				
**MCs**	**NCs**	**CSF Aβ42, T-tau, P-tau, Aβ42:Aβ40 ratio or plasma Aβ42 in MCs vs NCs**	**Comment**	**Ref**
88	40	↓ Aβ42 and ↑ T-tau in CSF, 10 and 15 years before expected symptom onset, respectively. ↑ Aβ42 in plasma 15 years before expected symptom onset.	A linear model of biomarker levels in relation to time to expected onset of symptoms was created from cross-sectional data, and used for comparisons of biomarker levels at various points in time. According to this model, CSF Aβ42 seemed to decrease over 25 years prior to expected symptom onset.	[11]
10	10	↑ Aβ42 and ↑Aβ42: Aβ40 ratio in CSF and ↑ Aβ42 in plasma 20 years before expected onset of MCI. → T-tau and → P-tau in CSF.	MCs had also elevated plasma Aβ42: Aβ40 ratios.	[12]
9	5	↓ Aβ42, ↑T-tau and ↑P-tau in CSF 17 years before expected dementia diagnosis.	CSF Aβ42 levels were negatively correlated with age in MCs.	[13]
6	6	↑T-tau, ↑ P-tau, ↓ Aβ42: Aβ40 ratio in CSF and → Aβ42 in plasma 11 years before expected AD diagnosis. A trend towards ↓ Aβ42 in CSF (p = 0.53) was also seen.		[14]
5 (CSF), 8 (plasma)	4 (CSF), 9 (plasma)	↑T-tau, ↑ P-tau in CSF and ↑ Aβ42 in plasma 13 years before expected AD diagnosis.	↑ Aβ42:Aβ40 ratios in plasma among MCs were also seen.	[15]

*Aβ* = amyloid β; *AD* = Alzheimer’s disease; *CSF* = cerebrospinal fluid; *MC* = mutation carrier; *NC* = non-carrier; *P-tau* = hyperphosphorylated tau, *Ref* = reference; *T-tau* = total tau.

**Table 2:** Summary of preclinical studies on patients with sporadic AD

**Sporadic AD**						
**Subjects**	**Developed dementia**	**Mean age (years)**	**Follow-up time (years)**	**CSF Aβ42, T-tau, P-tau, Aβ42:Aβ40 ratio or plasma Aβ42**	**Comment**	**Ref**
55	4	73	8	CSF T-tau was cross-sectionally correlated with MMSE score. CSF Aβ42 was correlated with future MMSE and change in MMSE.	There were only women in this study.	[16]
61	13 (had a CDR > 0)	75	3-4	No significant results for Aβ42, T-tau, P-tau in CSF.	The main outcome was to predict conversion from CDR 0 to a higher CDR. The CSF T-tau:Aβ42 and P-tau:Aβ42 ratios significantly predicted this.	[17]
51	0	73	3	↓ Aβ42 in CSF predicted cognitive decline. No significant results for T-tau or P-tau in CSF.	Cognition was evaluated with memQoL and MMSE. The combination of CSF Aβ42 and P-tau predicted cognitive decline with increased accuracy compared with Aβ42 alone.	[18]
37	0	73	4.5	↓ Aβ42 CSF at follow-up correlated with worse cognition. Individuals with the most substantial longitudinal decrease of Aβ42 or increase of P-tau in CSF performed worse on cognitive test than the others. No significant results for CSF T-tau and plasma Aβ42.	Cognition was evaluated with ADAS-cog and AQT.	[19]
43	0, but 4 developed MCI	Around 69^a^	3.5	Aβ42 and T-tau in CSF were used for predicting MCI.	Individuals were dichotomized into having a high or normal CSF T-tau/Aβ42 ratio. Individuals with a high ratio were more likely to develop MCI on follow-up.	[20]
35	7	85	3	↓Aβ42 in CSF at baseline in patients that developed dementia. The CSF Aβ42:Aβ40 ratio showed a tendency to be lower (p = 0.068) in individuals that developed dementia.	CSF Aβ40 was lower in patients with dementia at baseline, but could not predict dementia in the cognitively normal.	[21]
127	2, and 11 developed MCI	60	4	↓Aβ42, ↑T-tau and ↑ P-tau in CSF among progressors.	All patients had subjective complaints at baseline. CSF Aβ42 alone was the strongest predictor for clinical progression.	[22]

*Aβ* = amyloid β; *AD* = Alzheimer’s disease; *ADAS-cog* = Alzheimer Disease Assessment Scale–cognitive subscale; *AQT* = A Quick Test of Cognitive Speed; *CDR* = Clinical Dementia Rating; *CSF* = cerebrospinal fluid; *MCI* = mild cognitive impairment; memQoL = the memory item of the Quality-of-Life Alzheimer’s disease instrument; *MMSE* = mini-mental state examination; *P-tau* = hyperphosphorylated tau, *Ref* = reference; *T-tau* = total tau.

^a^Not stated specifically for these subgroups.

Longitudinal studies have correlated baseline levels of the core biomarkers in cognitively normal individuals with decrease in cognitive function or development of MCI or AD. CSF levels of Aβ42 alone [16] or in combination with T-tau [17] or P-tau [18] have been associated with future development of cognitive impairment in individuals that were cognitively normal at the time of LP. In another study, healthy older adults were examined and lumbar punctured at baseline and after a four year follow-up [19]. It was found that low levels of Aβ42 at follow-up were associated with worse performance on cognitive tests. Decreasing levels of Aβ42 and increasing levels of P-tau were also associated with worse cognitive performance. One study including healthy controls from the Alzheimer Disease Neuroimaging Initiative (ADNI) showed that individuals with low CSF Aβ42 levels displayed significantly higher rates of brain atrophy over one year than individuals with higher Aβ42 levels [23]. Increased T-tau:Aβ42 ratio [20] or low levels of CSF Aβ42 [21] have been found to predict conversion to MCI in cognitively normal or AD in non-demented elder individuals, respectively. One study on patients with subjective complaints found that CSF Aβ42 alone was superior to T-Tau, P-tau or a combination of all three biomarkers in predicting progression to MCI or AD [22]. However, there are yet no prospective long-term studies that have investigated whether CSF biomarkers can predict development of AD in healthy elderly individuals within 10–20 years. Such studies will be vital to be able to determine whether the majority of healthy individuals with low CSF Aβ42 (alone or in combination with tau) will indeed develop AD dementia within 1–2 decades.

In this context, it is important to remember that several of the longitudinal studies mentioned above are in fact cross-sectional in respect to the biomarker data, with one set of baseline biomarker measures correlated to longitudinal clinical data. This is not the best study design to examine temporal dynamics in biomarker changes; the sensitivity of the analytical techniques may determine when a biomarker appears positive. A very sensitive method for one marker as compared to a less sensitive method for another marker may give a false imprecision that one pathological change precedes the other. Therefore, we now must validate the hypotheses and models mentioned above in true longitudinal studies with repeated biomarker measurements and clinical assessments over time in the same individuals.

### Biomarkers in MCI

The risk for patients with MCI to develop AD during a 4.5-year follow-up has been found to be roughly tripled compared to cognitively healthy controls [24]. However, MCI is a heterogeneous disorder, and people suffering from it may progress to other dementias than AD, including vascular dementia (VAD), frontotemporal dementia (FTD) and Lewy body dementia (DLB), or remain relatively stable and decline cognitively as in normal aging [25]. The combined pattern of low levels of Aβ42 together with high levels of T-tau and P-tau in CSF can accurately discriminate incipient AD from patients with stable MCI [26]. Since the annual progression rate from MCI to AD is around 10-15% [27], it is important to have long follow up periods to identify late converters. One large study found that CSF levels of Aβ42, T-tau and P-tau among MCI patients could predict progression to AD with good accuracy after a median follow-up period of 5.2 years [28]. The combination of T-tau and Aβ42 gave a sensitivity of 95% and a specificity of 83%. The study population was evaluated again after an extended follow-up, now with a median of 9.2 years since baseline [29]. An additional set of patients from the MCI group had now converted to AD. The ratio of Aβ42:P-tau at baseline predicted development of AD within 9.2 years with sensitivity and specificity around 85-90%. Patients who converted within 5 years had significantly higher levels of T-tau and P-tau than the patients that converted within 5–10 years, while the level of Aβ42 was similar. A limitation of that particular study was however that the cutoff levels for the CSF biomarkers were established in the same cohort of patients that was then used to evaluate the diagnostic performance of the biomarkers to detect incipient AD. In some other studies, the cutoffs for Aβ42, T-tau and P-tau have been determined when comparing controls with patients with AD and then these cut offs have been applied in cohorts with MCI. Using this approach Hertze et al. found that different combinations of Aβ42, T-tau and P-tau could predict AD over 5 years with a sensitivity of 85-90% and specificity of 71-82% [30]. In a cohort from the Development of screening guidelines and criteria for predementia Alzheimer’s disease (DESCRIPA) study, 79% of the patients with amnestic MCI had an abnormal CSF Aβ42:T-tau ratio, and this profile was associated with progression to AD dementia [31]. A baseline AD-like profile of CSF T-tau:Aβ42 was present in 89% of MCI patients from ADNI that developed AD within one year [32].

A large multicenter study tested the ability of the core CSF AD biomarkers to predict incipient AD in patients with MCI [33]. The combination of Aβ42:P-tau ratio and T-Tau rendered a sensitivity of 83% and a specificity of 72% for progression to AD. The somewhat low specificity in this study may be caused by the short follow-up time of only around 2 years. Also, the biomarker cutoffs were determined in an independent cohort. This gives a lower, but truer, diagnostic accuracy. Further, lack of standardization of pre-analytical and analytical conditions between the included centers may have contributed.

The potential of the core biomarkers under standardized settings has proven to be very good. Their diagnostic performance in a homogeneous mono-center population was excellent, achieving an area under the receiver operating curve of 0.97 in the discrimination of patients with AD or patients with MCI who converted to AD against stable MCI and controls [34].

Indeed, for the biomarkers to be used clinically, standardization of pre-analytical and analytical factors is needed. This requires identification of these factors followed by harmonization between laboratories. The Alzheimer’s Association quality control program was created with the purpose of facilitating the worldwide use of CSF AD biomarkers in the clinic and research and is open for any laboratory that uses a commercially available assay for CSF Aβ42, T-tau or P-tau [35]. In the test rounds that have taken place so far, the coefficient of variation among the contributing labs has been around 15-25% [36]. Currently, only monitoring is conducted, but further on, active interventions will be crucial. To that end, several standardization efforts are in progress, e.g., the Global Biomarker Standardization Consortium, the CSF-Proteins Working Group of International Federation of Clinical Chemistry and Laboratory Medicine and the BIOMARKAPD project of the EU Joint Programme in Neurodegenerative Disease Research [36].

### Biomarkers in AD with dementia

CSF levels of T-tau are about 300% higher in AD patients than control subjects [37]. The biomarker is not specific for AD and increased levels can be found in the CSF of patients who have suffered from acute stroke [38] or head trauma [39], and very high levels are seen in patients with Creutzfelt-Jakob disease [40]. Since it is a non-specific marker for axonal and neuronal degeneration, non-AD dementias may also give elevated CSF T-tau levels [41]. In the differential diagnostics against other dementias, P-tau may have a greater value since it is more AD-specific [42]. Elevated P-tau levels have been found in patients with AD compared with patients suffering from FTD and VAD [43], DLB [43,44] and Parkinson disease with dementia [44]. P-tau differentiated between patients with AD and patients with FTD and DLB, with specificities of 92 and 64%, respectively [43]. However, a large study on patients with AD as well as DLB, FTD and VAD showed that an AD biomarker profile was present in a substantial part of the non-AD patients [45]. Potential reasons for this overlap may be misdiagnosis or the presence of mixed dementias. Future autopsy studies will hopefully shed light on this issue.

The levels of P-tau in CSF *ante mortem* correlate with the amount of neurofibrillary tangles and hyperphosphorylated tau in the brain *post mortem*[46,47]. A study of cortical biopsies from living patients with normal pressure hydrocephalus showed a correlation between the amount of hyperphosphorylated tau in the biopsies and P-tau levels in CSF [48]. The same study also found that presence of cortical amyloid plaques was associated with lower levels of CSF Aβ42. The lower CSF levels of Aβ42 in AD patients compared with controls are believed to be caused by its accumulation in senile plaques. Indeed, autopsy studies have reported correlations between plaque load and reduced Aβ42 in lumbar CSF *ante mortem*[46] and ventricular CSF *post mortem*[49]. Using positron emission tomography several studies found an inverse correlation between brain amyloid load and CSF Aβ42 [50-52].

The combination of these biomarkers can accurately distinguish AD patients from controls, with sensitivity and specificity over 80% [26,41]. One study that examined the performance of these biomarkers in AD patients in different ages found that although diagnostic accuracy decreased somewhat with age, the positive and negative predictive values of the biomarkers combined were stable enough for the biomarkers to be used in old patients [53]. Their longitudinal stability in AD patients has also been evaluated. Most studies have found that levels of Aβ42 and P-tau remain unaltered over time [54-57] while available data for T-tau is inconclusive [58]. Some studies report no temporal alteration in T-tau levels in AD patients, while others have found an increase. However, high levels of T-tau and P-tau seems to be associated with increased disease intensity and a more rapid disease progression [59-61].

### Measurement of core AD biomarkers

There are various assays for the three core AD CSF biomarkers and it is important to bear in mind what they actually measure. One of the most common assays for Aβ42 is an enzyme-linked immunosorbent assay (ELISA) specifically constructed to measure Aβ containing both the 1st and 42nd amino acids of the protein [62], but there are also assays that are C-terminally end-specific but use N-terminal antibodies that allow for measurement of N-terminally truncated Aβ42 fragments in addition to Aβ1-42 [63]. Most data suggest that these assays measure free monomeric Aβ42 but they correlate well with a selected reaction monitoring-based mass spectrometry method for total Aβ42 [64]. Tau exists in several isoforms and may be phosphorylated at different residues. The most common ELISA for T-tau detects all isoforms of tau, independently of phosphorylation state [65]. The two most common ELISAs for P-tau measure tau that is phosphorylated at residue 181 or 231 [66][67][68], and the diagnostic performance is similar between these assays [43]. A multiparameter assay based on xMAP® technology was developed for simultaneous measurement of Aβ42, T-tau and P-tau [68]. Although it gives different absolute values than the corresponding ELISAs, correction factors can be used for direct comparisons.

### CSF and imaging biomarkers combined

Even though imaging biomarkers are beyond the scope of this review we would like to point out some studies where CSF and imaging biomarkers have been used together. The combination of CSF and imaging biomarkers can provide increased diagnostic accuracy compared to using either modality alone. Shaffer et al. found this to be the case when using whole-brain MRI, fluorine 18 fluorodeoxyglucose (FDG) PET and CSF T-tau, P-tau and Aβ42 to predict conversion from MCI to AD [69]. Westman et al. showed that the combination of CSF Aβ42, T-tau and P-tau with MRI-generated regional subcortical volumes and cortical thickness measures gives a better classification of MCI, AD and controls at baseline than using either biomarker alone [70]. The prediction of incipient AD in MCI subjects was also improved by using the two biomarker modalities together. Vos et al. found that the CSF Aβ42:T-tau ratio increased the predictive accuracy for MCI patients with normal and abnormal hippocampal volumes on MRI that later developed AD [71]. MRI only provided increased diagnostic accuracy in patients with a normal CSF test. Other studies have found that CSF T-tau, P-tau, Aβ40 and/or Aβ42 and atrophy of the medial temporal lobe on MRI provides independent information when discriminating between AD patients and controls [72], and between patients with stable MCI and MCI patients that develop AD [73]. Further studies need to elucidate which imaging methods that are most beneficial to use, and the correct time point to implement them, in relation to CSF biomarkers.

### Candidate biomarkers

The search for new AD biomarkers may be facilitated by the core CSF AD biomarkers. By including patients with signs of an AD pathological process and ensuring that control subjects lack this profile, future biomarker studies may be more successful. Results from earlier studies may, for instance, have been clouded by the presence of non-symptomatic AD patients in the control groups. A recent study on the effect size of the major susceptibility gene for AD, *APOE* ϵ4, supports this reasoning; when disregarding clinical information on patients with cognitive impairment and simply grouping them on the basis of CSF tau and Aβ markers, the association of *APOE* ϵ4 with AD was twice as strong as compared to when classifying patients according to clinical status [74].

The potential uses for AD biomarkers are many. Besides from diagnostics, CSF biomarkers may be utilized for prognosis, assessing disease progression, developing treatments, monitoring treatment effects and studying disease mechanisms. Several studies have investigated the AD biomarker potential of various analytes related to Aβ.

#### BACE1

For Aβ to be produced, the amyloid precursor protein (APP) is cut by two different enzymes, β-secretase and γ-secretase. The major β-secretase in the brain is called β-site APP cleaving enzyme-1 (BACE1). Several studies have investigated the levels of CSF BACE1 activity in patients with MCI and AD, but the results are not univocal. Three small studies found that patients with AD had increased BACE1 activity compared with non-demented controls [75,76] or patients with other dementias [77]. One study found higher activity in patients with MCI and dementia due to AD compared with controls [63]. Another study found significantly elevated BACE1 activity in patients with MCI but not with AD when comparing with controls [78]. One study failed to show any differences in BACE1 activity between MCI and AD patients compared with controls [79]. However, when patients with a pathologic profile of the core AD biomarkers were compared with controls with a normal biomarker pattern, a significant elevation of BACE1 activity was found in the patient group. The MCI patients contributed the most to this elevation. Finally, a large study did not find any differences in activity between AD patients and controls [80]. When the AD patients were stratified into mild and moderate-severe AD, an increased BACE1 activity could be seen in the group with mild AD compared the more affected AD patients and controls. These studies indicate that the activity of BACE1 may be mildly elevated in the early stages of AD, but the diagnostic usability of the biomarker seems limited. It may however prove to be valuable in clinical trials of BACE1 inhibitors.

#### sAPPα/sAPPβ

The levels of sAPPα and sAPPβ correlate very well in AD patients as well as controls [63]. Several studies have failed to show any differences in the levels of these biomarkers between AD patients and controls [30,34,63,80,81]. One study found higher levels of sAPPβ in MCI patients compared with controls [81], and another that MCI patients that upon follow up developed AD had higher levels of sAPPβ than patients that didn’t [82]. However, Hertze et al. found no differences in sAPP levels in MCI patients with incipient AD compared with stable MCIs or patients that developed other dementias [30]. Studies that compared patients with MCI or dementia that had a pathologic core CSF AD biomarker profile with controls with a normal profile found that the former group had increased levels of sAPPα and sAPPβ, but there were large overlaps between the groups [83-85]. The diagnostic value of sAPPα and sAPPβ appears limited. Yet, the proteins may be used for studying effects on the APP metabolism in clinical trials [5].

#### Aβ oligomers

Oligomers of Aβ may be more toxic than fibrillar Aβ aggregates [86]. They can inhibit hippocampal long-term potentiation *in vivo*[87], and cause abnormal tau phosphorylation and neuritic dystrophy [88-90]. A relatively large number of assay formats to measure Aβ oligomers in CSF have been published. These papers have shown that the CSF level of Aβ oligomers is very low, probably less than 1% of total Aβ levels, and thereby very difficult to quantify in a reliable manner.

In AD patients, elevated Aβ oligomer levels have been found in brains [91,92] and CSF [93-96]. However, not all studies have found altered levels in AD patients versus controls [97,98], but the latter of these found a negative correlation between oligomer levels and cognitive status, indicating a potential use for assessing disease stage in AD patients. One study found elevated levels of oligomers in human and mouse brains, but not in CSF [99]. Handoko et al. found that CSF oligomer levels increased with age and correlated with levels of T-tau in cognitively normal older adults. Also, increased levels were found in cognitively normal subjects with a biomarker profile indicating impending AD [100]. Except for technical difficulties to measure minute amounts of Aβ oligomers in CSF samples, it is also possible that different assays measure different variants of Aβ oligomers, which might explain the divergent results. The studies provide little characterization of what the various assays are even measuring.

#### Other candidate markers

In addition to the amyloid cascade hypothesis, genetics points to at least three other pathways that may be of relevance in AD pathogenesis: (i) the innate immune system, (ii) cholesterol metabolism and (iii) endosomal vesicle recycling. Better biomarkers for these processes may give us better tools to assess the importance of aberrations in these pathways and to subgroup AD patients according to which pathway is most abnormal.

Candidate biomarkers may help us to better characterize the ongoing pathologic processes in the AD brain. One example of this is the inflammatory biomarker YKL-40, which is secreted by activated microglia [101]. Studies have found increased levels of CSF YKL-40 in AD patients, as a sign of microglial activation [102-104]. Although Craig-Shapiro et al. suggested that YKL-40 is secreted by astrocytes, data from cell cultures and immunohistochemistry show that microglia, rather than astrocytes, are responsible for the main production of YKL-40 [101]. In an experimental setting, Simard et al. provided further insight into the role of microglia in AD by showing that blood-derived microglia can prevent the formation of or eliminate amyloid plaques in transgenic mice [105]. There are several other interesting candidate biomarkers, such as visinin-like protein-1 [106], neurogranin [107] and F2-isoprostane [108].

### Blood biomarkers

Since blood is more easily accessible than CSF, finding reliable blood biomarkers for AD is desirable. Due to the blood–brain barrier, the concentration of brain-derived proteins in the blood is lower than in the CSF, which makes this task a challenge. Further, brain-derived proteins are diluted in the large plasma volume and may also get degraded. Recent studies point to important differences in the regulation of, e.g., tau concentrations in CSF as compared to blood. In patients with hypoxic brain injury following cardiac arrest, tau is rapidly released into the bloodstream but effectively (within 24 hours) cleared in patients with good neurological outcome [109]. In contrast, CSF tau levels stay elevated for many weeks following an acute neurological insult [38]. Further, tau levels are clearly elevated in CSF from patients with Alzheimer’s disease, but less so in the corresponding plasma samples; measurements of tau in these two compartments do not correlate [110].

Many studies have examined the association of plasma Aβ40 and Aβ42 levels with incipient AD, but the results are not clear-cut. Some studies have found that elevated Aβ40 [111,112] or Aβ42 [] levels predicted development of AD, while others have found the opposite [115] or no associations [116,117]. Some studies found that a low Aβ42:Aβ40 ratio predicted future AD [111,118,119] while others report an elevated ratio [112,113] or no significant difference [116] in the group with incipient AD compared with subjects that didn’t develop AD. Recently, a meta-analysis over the literature indicated that a low Ab42:Ab40 ratio could predict development of AD, but no such association was found for the individual peptides [120]. More research in the area is needed. Cross-sectional studies that have compared Aβ levels in AD patients and controls have mostly found similar group levels [121]. In one study, high levels of Aβ42 was associated with a more rapid rate of cognitive decline among AD patients [122]. Recent studies have, by measuring the levels of several plasma analytes simultaneously, found biomarker patterns that successfully differentiated AD patients from controls [123,124] or were associated with MCI or AD [125]. However, some of these finding have been hard to reproduce in independent studies [126,127]. Other studies have found that plasma analytes such as desmosterol [128], transthyretin [129], clusterin [130], chitinase 3-like 1 protein [131] and matrix metalloproteinase 2 [132] may be associated with AD. This needs to be corroborated in further studies. Several of these biomarkers have also been associated with AD when measured in CSF.

### Limitations

The major and inherent limitation of fluid biomarkers is the lack of anatomical precision in the measurements. Another limitation is the lack of assay standardization. Different assays for, e.g., Aβ42 correlate but give different absolute concentrations of the protein. This prevents from the use of global reference limits and diagnostic cut-points. Intense work is currently ongoing to achieve better assay standardization through the establishment of reference measurement procedures for tau and Aβ42 and to validate these in multi-center settings [36].

There is also a paucity of studies that connect human and experimental work. Although the biomarker CSF T-tau has been extensively studied there are some basic aspects that still are unknown. What does steady-state concentrations of tau among healthy individuals reflect; is it a low grade neurodegeneration or perhaps normal neuroaxonal plasticity? It is also not known why some tau pathologies, e.g. progressive supranuclear palsy, typically have normal levels of tau and why CSF levels of tau remain largely unaltered with progression of AD. An explanation to the latter could be that the levels of tau are more dependent on the rate of neurodegeneration than the state, but questions like these warrant translational studies spanning from models to human disease.

## Conclusions

Three core CSF AD biomarkers have been evaluated in a great number of studies and may provide valuable information in all stages of AD. Due to differences in analytic results between studies and laboratories, standardization of analytic procedures is essential for the use of these biomarkers in clinics and in research. Apart from the core biomarkers, there are several candidate CSF and blood biomarkers in the pipeline, but they need verification in further studies.

## Competing interests

The authors declare that they have no competing interest.

## Authors’ contributions

CR drafted the manuscript. OH, KB and HZ revised the manuscript for important intellectual content. All authors read and approved the final manuscript.
